# Benefits of Volunteering for Middle-Aged and Older Adults: Comparison Between Types of Volunteering Activities

**DOI:** 10.1093/geroni/igaa057.1305

**Published:** 2020-12-16

**Authors:** Edwin K H Chung, Alfred H K Lam, Dannii Yeung, Ka Hung Edwin Chung

**Affiliations:** City University of Hong Kong, Hong Kong, China

## Abstract

More middle-aged and older adults are often encouraged to volunteer (Gray et al., 2012). However, the effects of various volunteering activities on physical, psychological and cognitive health remain unknown. This study thereby aims to investigate such effects and the moderating effect of age in such associations. Data of 501 middle-aged and older Hong Kong Chinese adults (Mage = 53.06, SD = 4.55; and Mage = 70.46, SD = 7.34, respectively; range = 45 – 96) from a survey on Adult Development and Aging were analysed. Engagements in instrumental (e.g., food preparation, fundraising) and cognitively demanding volunteering (e.g., counselling, mentoring), hand-grip strength, life satisfaction, depressive symptoms, and cognitive functioning were measured. Factorial ANOVA revealed significant main effects of age group and volunteering type (F = 29.71, and F = 3.96, respectively, ps < .001), and an interaction effect of age and volunteering type (F =1.80, p = .03) on health outcomes. Comparisons among the four volunteering types (no volunteering, instrumental volunteering, cognitively demanding volunteering, and both types) revealed that individuals engaging in cognitively demanding volunteering had better hand-grip strength, life satisfaction, and cognitive functioning, and lower depressive symptoms than those who engaged in instrumental volunteering (all ps < .05). The health outcomes of instrumental volunteering were even worse than those who did not volunteer at all. These patterns were more prevalent in the middle-aged adults than in the older adults. Findings of this study indicated the beneficial effects of cognitively demanding volunteering, providing valuable directions for future programs on volunteering.

